# Supplementary material for: p21 maintains senescent cell viability under persistent DNA damage response by restraining JNK and caspase signaling
Source: EMBO J. 2017 Jun 12;36(15):2280–95. doi: 10.15252/embj.201695553 (PMC5538795; doi:10.15252/embj.201695553)
Supplement: Supplementary file 1 — Appendix [file EMBJ-36-2280-s001.pdf]

Appendix

Yosef et al,

p21 maintains senescent cell viability under persistent DNA damage response by restraining JNK and caspase signaling

Table of Contents

Appendix Figure Legends ……………………………………………...page 2

Appendix Table Legends ………………………………………………page 6

Appendix Figures …………………………………………………………page 7

Appendix Tables …………………………………………………………..page 17

Appendix Figure Legends

Appendix Figure S1. Effect of Etoposide on Primary BJ fibroblast. (A) Experimental design and reference time frame scheme. (B) Representative growth curves of growing (G) and Etoposide treated (DNA damage-induced senescent (DIS)) BJ fibroblasts. The time frame corresponds to the scheme presented in plot A. Each curve was performed at least twice, and each time point was determined in triplicate. (C) Pictures of growing and DIS BJ cells stained for SA-β-galactosidase activity at day 7 post etoposide treatment. (D) Quantification of BrdU incorporation (4 hours) in G and DIS cells transduced with sip21 or siCtrl, 4 days post siRNA washout; (E) Western blots of p16, p21 and p53 in G and DIS BJ cells. (F) Survival of DIS BJ cells, transduced with siRNAs targeting p21 or control siRNA three weeks following Etoposide treatment; the indicated time points are following siRNA washout. Data are presented as means ± SEM of 3 repeats, each performed in triplicate.

Appendix Figure S2. p21 knockdown reduces cancer senescent cells survival following DNA damage. (A) Pictures of growing (G) and DNA-damage induced senescence-like (DIS) H1299 lung cancer cells stained for SA-β-galactosidase (pH 6.0) activity at day 7 post etoposide treatment. Pictures are at the same magnification. (B) Immunofluorescence analysis of p-γH2AX in G and DIS BJ cells transduced with sip21 or siCtrl; representative images of DIS cells in at least two independent experiments are shown. (C) Cell cycle analysis of G and DIS H1299 cells; representative histograms of DAPI-Area of at least two independent experiments are shown. (D) Survival of G and DIS H1299 cells infected with small hairpin RNA (shRNA) targeting p21 (shp21) or control

shRNA targeting Luciferase (shLuci) prior to etoposide treatment. Western blots show p21 protein levels in the corresponding samples. Data are presented as mean±S.E.M of three repeats, performed in triplicates. Data was analyzed using Student's *t*-test. ***$P < 0.0005$.

Appendix Figure S3. Cell Cycle pathway from WikiPathways. (A) An overview of the series of events that takes place in a cell leading to its division and duplication**.** In Blue are the genes that their expression is altered following p21 knockdown only in DIS cells are depicted blue in the pathway map. (http://www.wikipathways.org/index.php/Pathway:WP179).

Appendix Figure S4. DNA damage response pathway from WikiPathways. (A) An overview of gene products, processes and changes in the DNA damage response pathway following p21 knockdown are depicted blue in the pathway map. (http://www.wikipathways.org/index.php/Pathway:WP707).

Appendix Figure S5. TGF-β signaling pathway from WikiPathways. (A) The interactions and intersections between canonical and non-canonical TGF-β signaling that are altered following p21 silencing in DIS cells are depicted blue in the pathway map.

(http://www.wikipathways.org/index.php/Pathway:WP366).

Appendix Figure S6. Focal adhesion pathway from WikiPathways. (A) Cell-matrix adhesions and interactions that are altered following p21 silencing in DIS cells are depicted in blue the pathway map.

(http://www.wikipathways.org/index.php/Pathway:WP306).

Appendix Figure S7. Hepatic stellate cell activation pathway from Ingenuity. (A) Predicted changes in the activation state of hepatic stellate cells (HSCs) in liver fibrosis following p21 knockdown are depicted in the pathway map. Green: downregulated genes; Red: upregulated genes.

(https://targetexplorer.ingenuity.com/pathway/ING/ING:1nilk#!/api/rest/v1/client/searchPathwayNodes?pathwayId=ING:1nilk&rows=0&facetLimit=5000&responseType=default).

Appendix Figure S8. Quantitative analysis of the distribution of γH2AX foci in G and DIS cells transduced with sip21 or siCtrl. ImageStreamX analysis of the distribution of numbers of γH2AX foci was performed on G and DIS cells transduced with sip21 or siCtrl 3 days after siRNA washout. DIS cells stained with the secondary antibodies only and DAPI served as a negative control. Average percent of positive cells in each sample was calculated. Data are presented as mean±S.E.M of three repeats. $^{**}P < 0.005$.

Appendix Figure S9. Partial rescue of DIS cell death following p21 knockdown by z-VAD-fmk. (A) Percentage survival of DIS BJ cells treated with siRNAs targeting p21 or control siRNA, with or without incubation with the cell-permeable, irreversible pan-caspase inhibitor z-VAD-fmk (z-VAD). Treatment with the inhibitor only partially rescues the cells from sip21-induced decrease in cell viability. The western blots show p21, cleaved PARP and caspase-3 proteins level following siRNA treatment. Data were presented as mean±S.E.M of three biological triplicates. Data was analyzed using Student's t-test. $^{*}P < 0.05$.

Appendix Figure S10. Schematic presentation of molecular events that occur in senescent cells following p21 knockdown.

Appendix Table Legends

Appendix Table S1, related to Figure 2: Cell cycle pathway altered genes. List of genes significantly changed in cell cycle pathway from WikiPathways following p21 silencing.

Appendix Table S2, related to Figure 2: DNA damage response pathway altered genes. List of genes significantly changed in DNA damage response pathway from WikiPathways following p21 silencing.

Appendix Table S3, related to Figure 2: TGF-β signaling pathway altered genes. List of genes significantly changed in TGF-β signaling pathway from WikiPathways following p21 silencing.

Appendix Table S4, related to Figure 2: Focal adhesion pathway altered genes. List of genes significantly changed in Focal adhesion pathway from WikiPathways following p21 silencing.

Appendix Figures

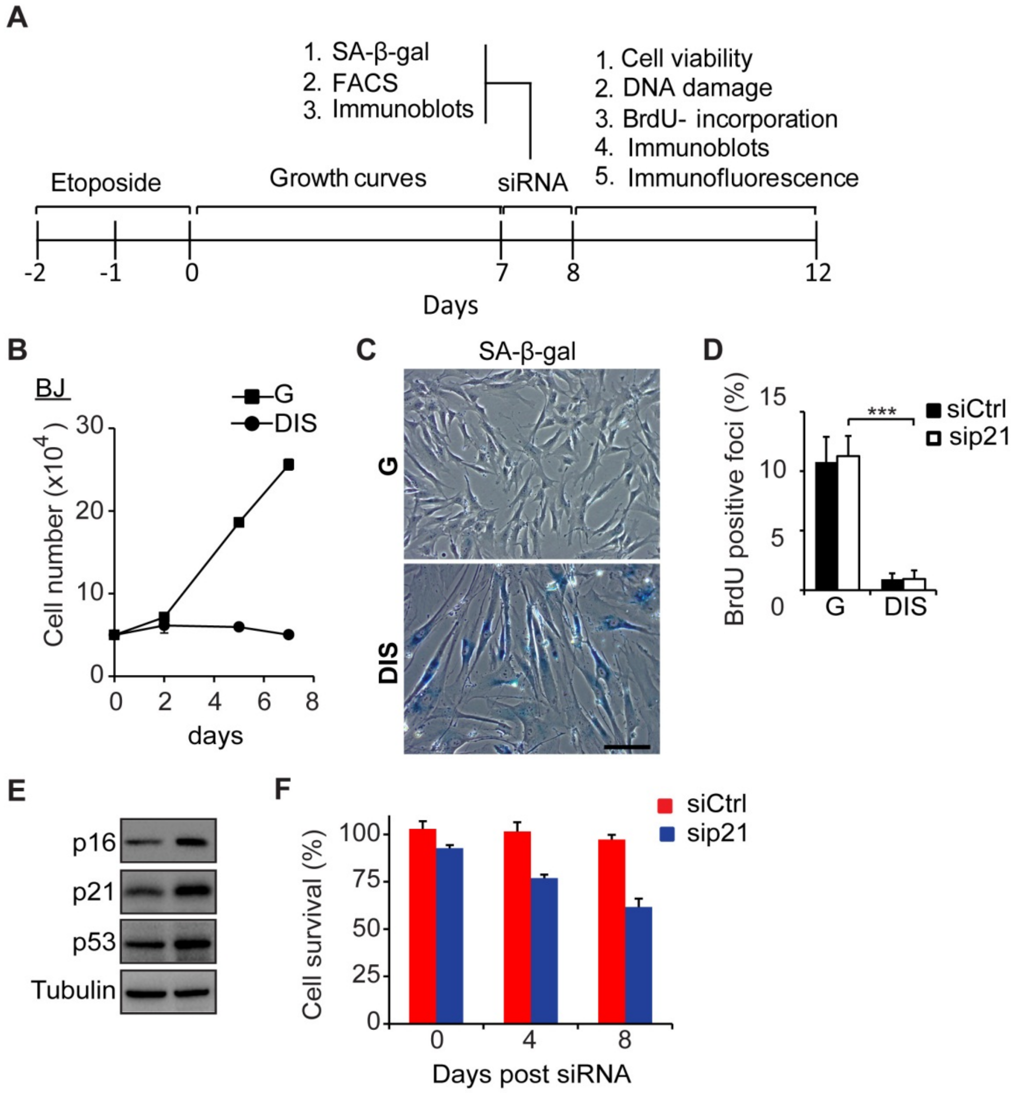

Appendix Figure S1

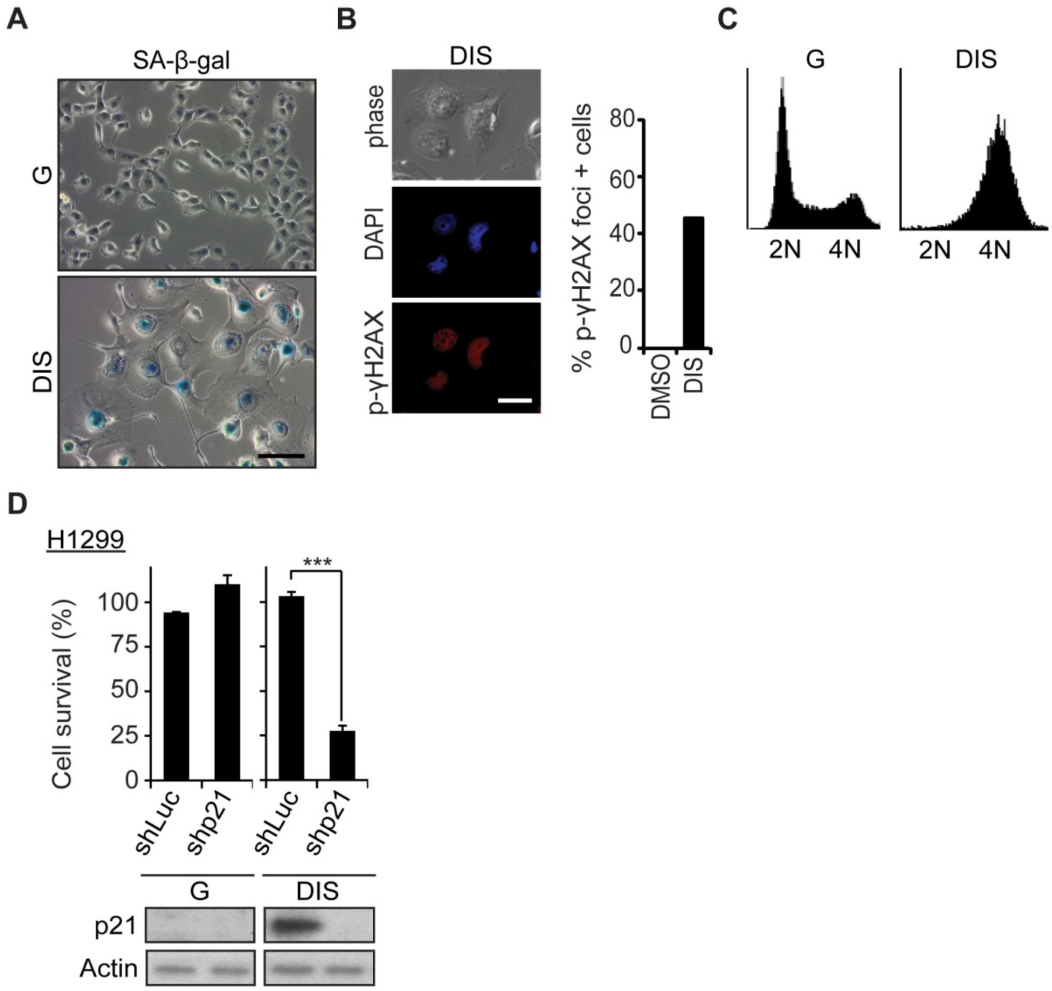

Appendix Figure S2

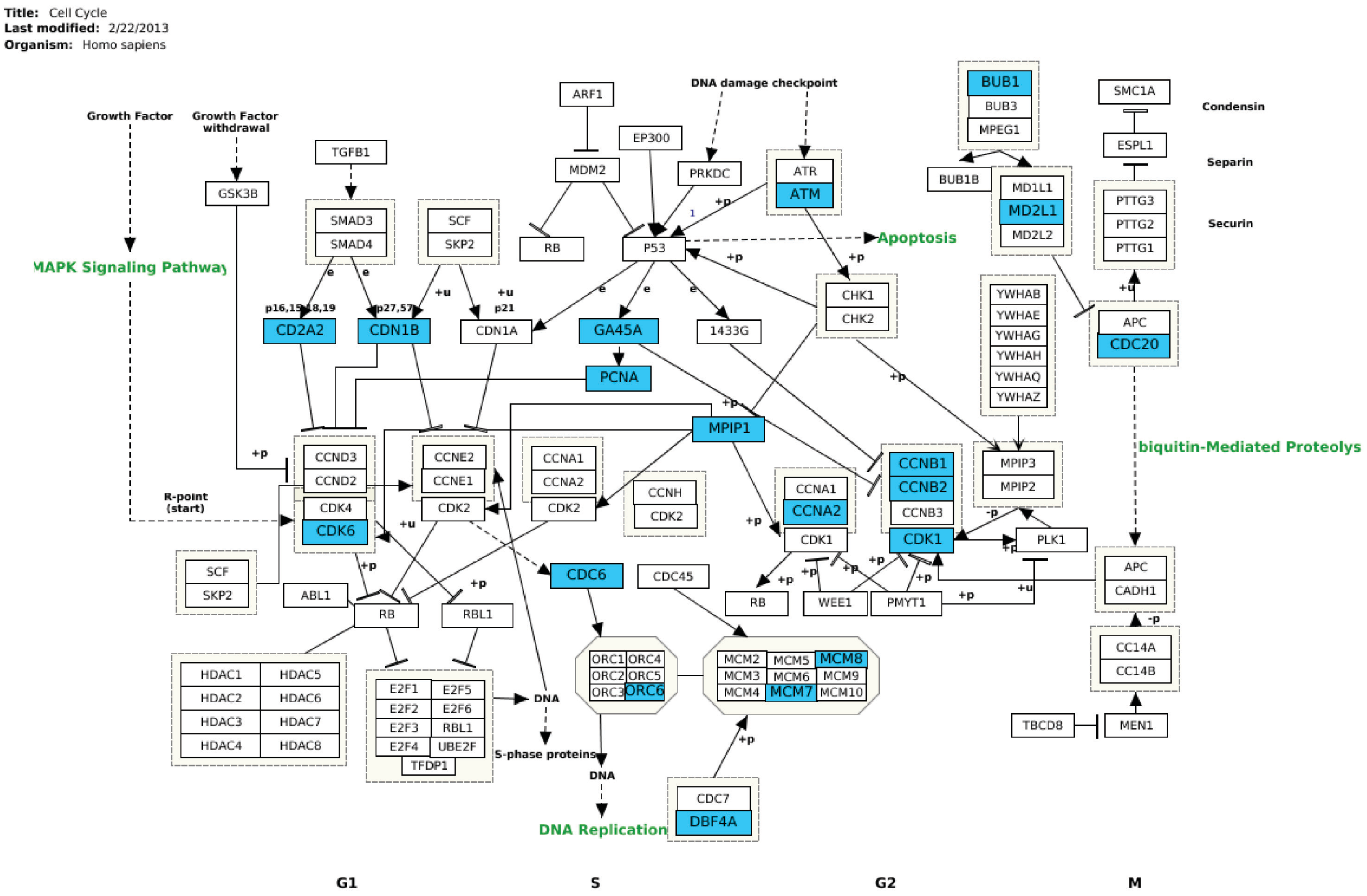

Appendix Figure S3

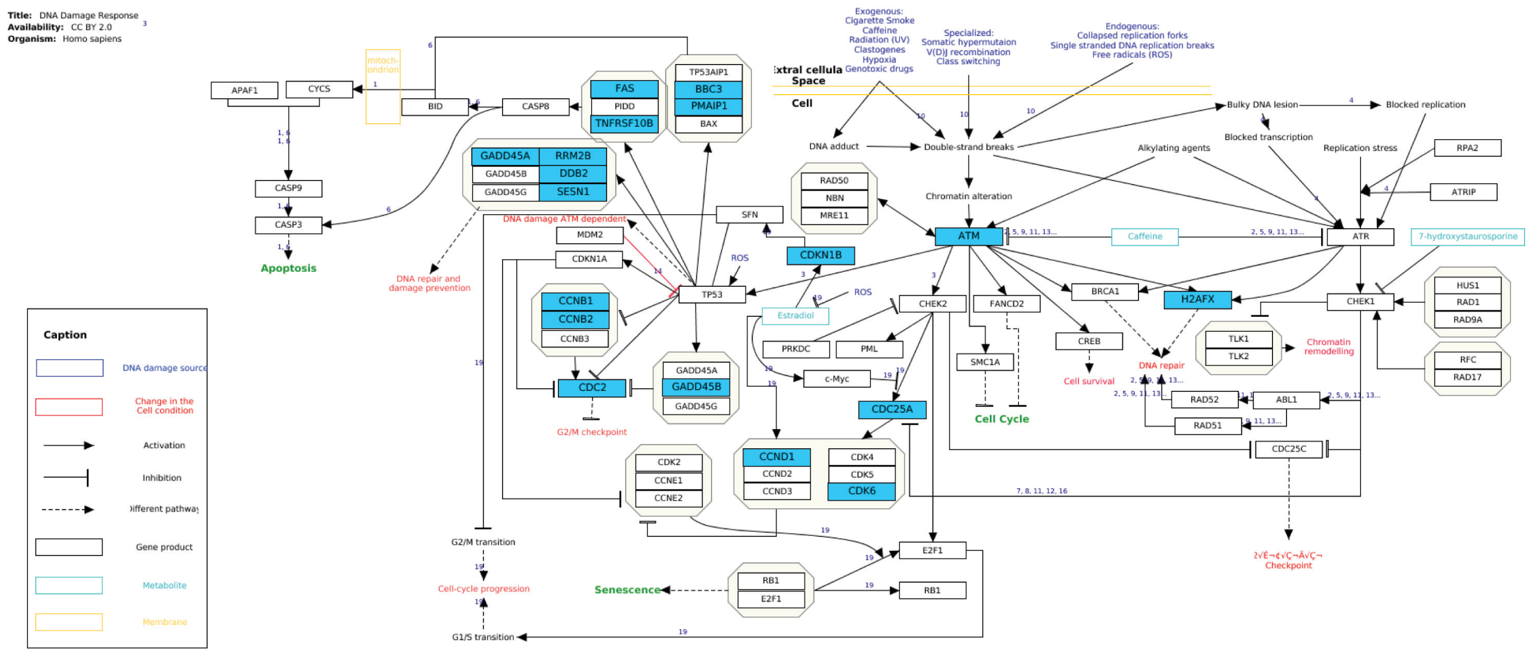

Appendix Figure S4

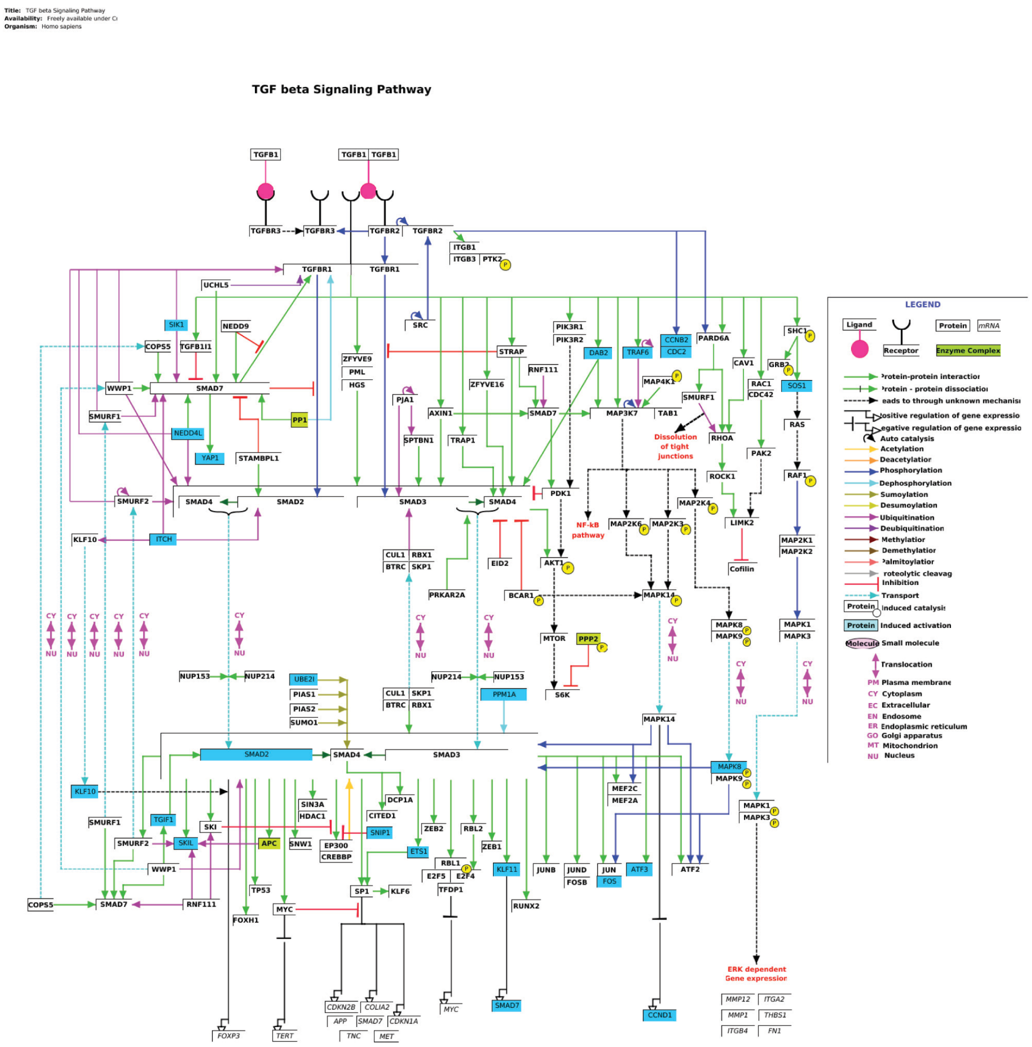

Appendix Figure S5

Title: Focal Adhesion 1-17
Organism: Homo sapiens

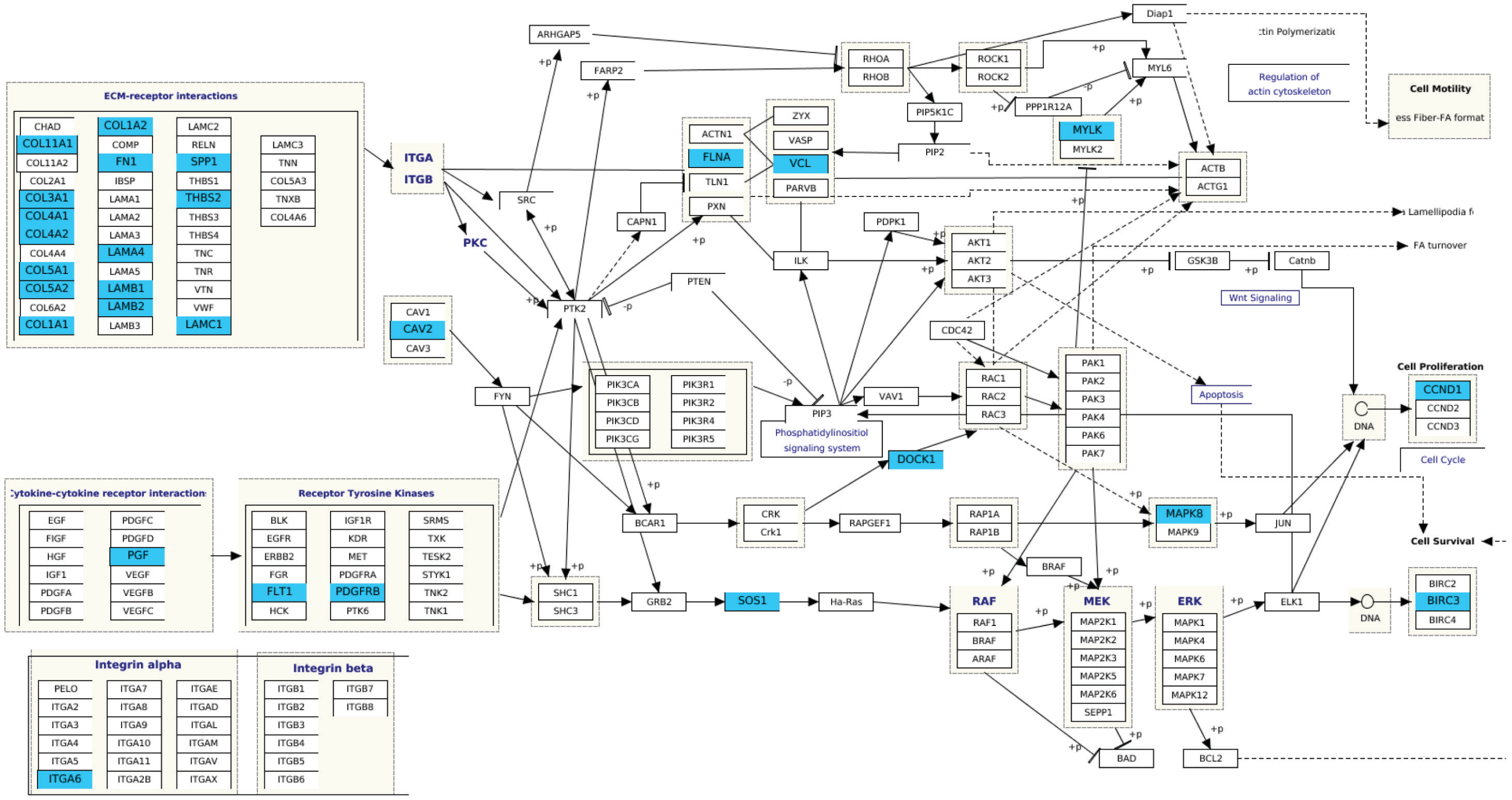

Appendix Figure S6

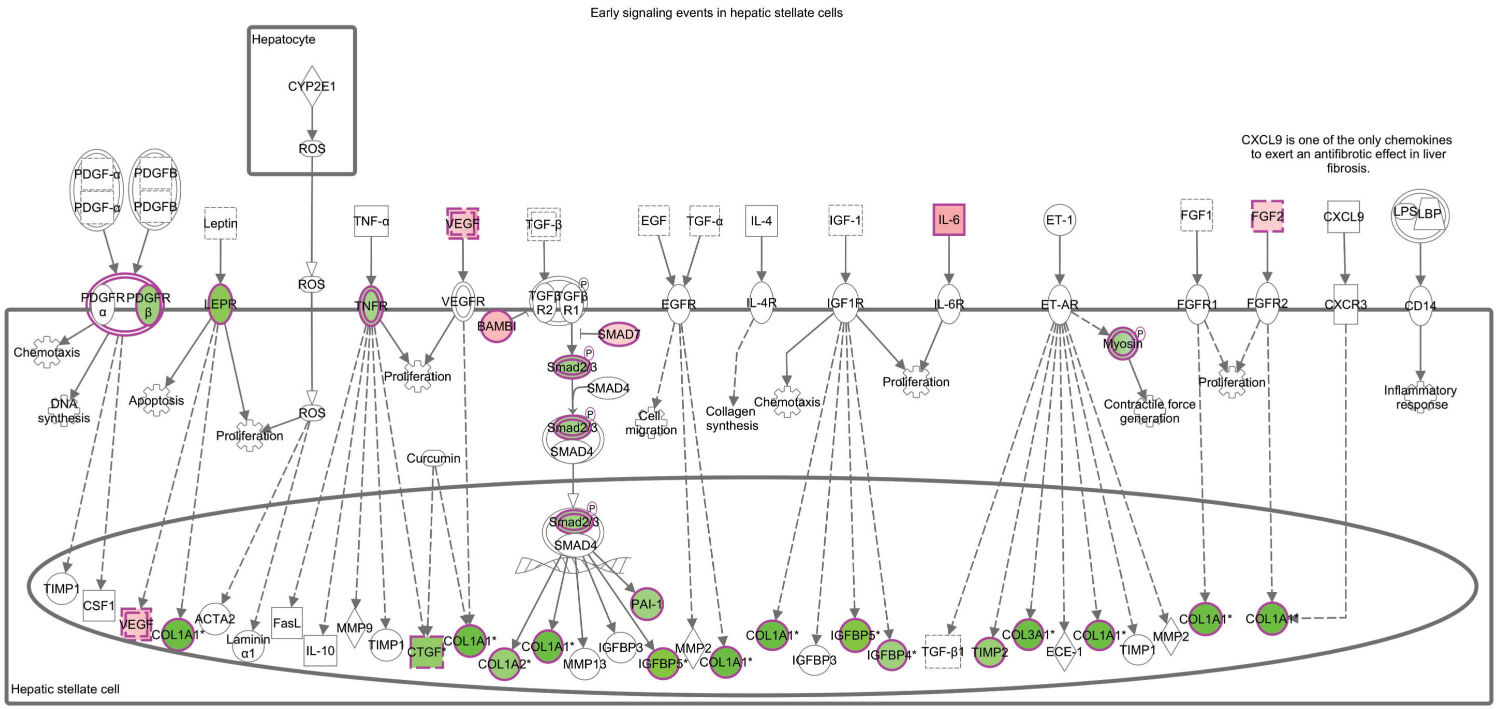

Appendix Figure S7

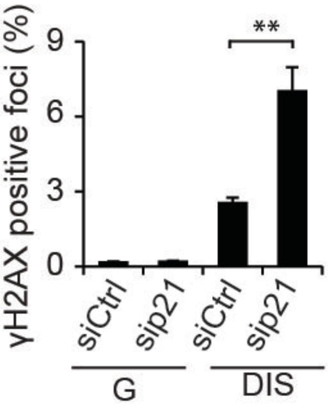

Appendix Figure S8

A

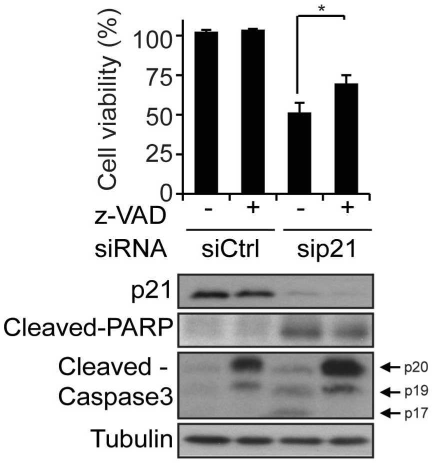

Appendix Figure S9

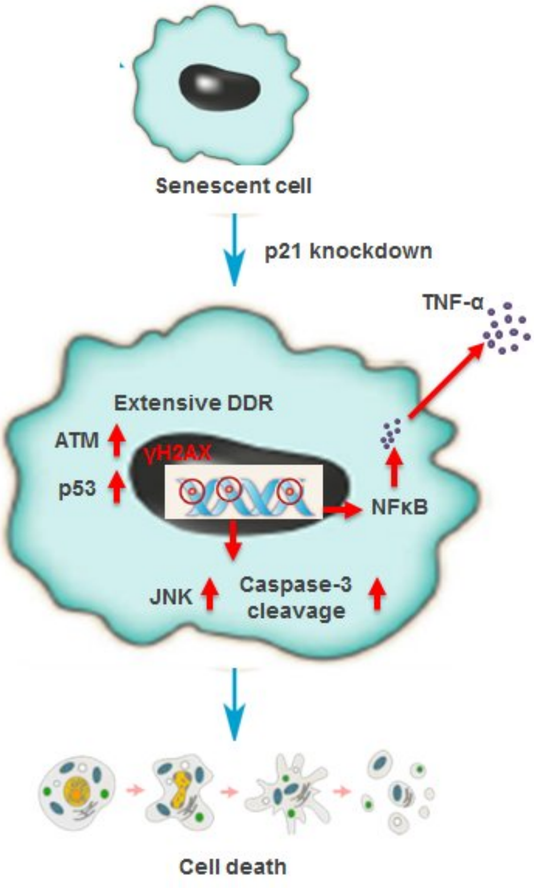

Appendix Figure S10

Appendix tables

Appendix Table S1.

Database:Wikipathways pathway Name:DNA damage response ID:WP707			
C=71; O=18; E=2.45; R=7.35; rawP=2.35e-11; adjP=1.88e-10			
Index	Gene Symbol	Gene Name	EntrezGene
1	DDB2	damage-specific DNA binding protein 2, 48kDa	1643
2	CDC25A	cell division cycle 25 homolog A (S. pombe)	993
3	TNFRSF10B	tumor necrosis factor receptor superfamily, member 10b	8795
4	CCNB1	cyclin B1	891
5	SESN1	sestrin 1	27244
6	CDK1	cyclin-dependent kinase 1	983
7	FAS	Fas (TNF receptor superfamily, member 6)	355
8	CCND1	cyclin D1	595
9	GADD45A	growth arrest and DNA-damage-inducible, alpha	1647
10	PMAIP1	phorbol-12-myristate-13-acetate-induced protein 1	5366
11	RRM2B	ribonucleotide reductase M2 B (TP53 inducible)	50484
12	GADD45B	growth arrest and DNA-damage-inducible, beta	4616
13	H2AFX	H2A histone family, member X	3014
14	BBC3	BCL2 binding component 3	27113
15	CDKN1B	cyclin-dependent kinase inhibitor 1B (p27, Kip1)	1027
16	ATM	ataxia telangiectasia mutated	472
17	CCNB2	cyclin B2	9133
18	CDK6	cyclin-dependent kinase 6	1021

Appendix Table S2.

Database:Wikipathways pathway Name:Focal Adhesion ID:WP306			
C=185; O=28; E=6.38; R=4.39; rawP=5.55e-11; adjP=3.70e-10			
Index	Gene Symbol	Gene Name	EntrezGene
1	LAMA4	laminin, alpha 4	3910
2	MAPK8	mitogen-activated protein kinase 8	5599
3	COL5A2	collagen, type V, alpha 2	1290
4	BIRC3	baculoviral IAP repeat containing 3	330
5	COL4A2	collagen, type IV, alpha 2	1284
6	LAMC1	laminin, gamma 1 (formerly LAMB2)	3915
7	FN1	fibronectin 1	2335
8	DOCK1	dedicator of cytokinesis 1	1793
9	SOS1	son of sevenless homolog 1 (Drosophila)	6654
10	THBS2	thrombospondin 2	7058
11	COL1A1	collagen, type I, alpha 1	1277
12	ITGA6	integrin, alpha 6	3655
13	COL11A1	collagen, type XI, alpha 1	1301
14	FLNA	filamin A, alpha	2316
15	COL3A1	collagen, type III, alpha 1	1281
16	PGF	placental growth factor	5228
17	CCND1	cyclin D1	595
18	SPP1	secreted phosphoprotein 1	6696
19	FLT1	fms-related tyrosine kinase 1 (vascular endothelial growth factor/vascular permeability factor receptor)	2321
20	MYLK	myosin light chain kinase	4638
21	COL5A1	collagen, type V, alpha 1	1289
22	LAMB1	laminin, beta 1	3912
23	LAMB2	laminin, beta 2 (laminin S)	3913

24	COL4A1	collagen, type IV, alpha 1	1282
25	COL1A2	collagen, type I, alpha 2	1278
26	VCL	Vinculin	7414
27	CAV2	caveolin 2	858
28	PDGFRB	platelet-derived growth factor receptor, beta polypeptide	5159

Appendix Table S3.

Database:Wikipathways pathway Name:TGF beta Signaling Pathway ID:WP366			
C=148; O=23; E=5.10; R=4.51; rawP=1.66e-09; adjP=8.30e-09			
Index	Gene Symbol	Gene Name	EntrezGene
1	MAPK8	mitogen-activated protein kinase 8	5599
2	TGIF1	TGFB-induced factor homeobox 1	7050
3	TRAF6	TNF receptor-associated factor 6, E3 ubiquitin protein ligase	7189
4	SNIP1	Smad nuclear interacting protein 1	79753
5	KLF10	Kruppel-like factor 10	7071
6	SOS1	son of sevenless homolog 1 (Drosophila)	6654
7	ITCH	itchy E3 ubiquitin protein ligase	83737
8	SMAD7	SMAD family member 7	4092
9	YAP1	Yes-associated protein 1	10413
10	ATF3	activating transcription factor 3	467
11	NEDD4L	neural precursor cell expressed, developmentally down-regulated 4-like, E3 ubiquitin protein ligase	23327
12	UBE2I	ubiquitin-conjugating enzyme E2I	7329
13	FOS	FBJ murine osteosarcoma viral oncogene homolog	2353
14	ETS1	v-ets erythroblastosis virus E26 oncogene homolog 1 (avian)	2113
15	CDK1	cyclin-dependent kinase 1	983
16	SKIL	SKI-like oncogene	6498
17	CCND1	cyclin D1	595

18	DAB2	disabled homolog 2, mitogen-responsive phosphoprotein (Drosophila)	1601
19	SMAD2	SMAD family member 2	4087
20	KLF11	Kruppel-like factor 11	8462
21	PPM1A	protein phosphatase, Mg2+/Mn2+ dependent, 1A	5494
22	SIK1	salt-inducible kinase 1	150094
23	CCNB2	cyclin B2	9133

Appendix Table S4.

Database:Wikipathways pathway Name:Cell cycle ID:WP179			
C=110; O=19; E=3.79; R=5.01; rawP=7.15e-09; adjP=2.86e-08			
Index	Gene Symbol	Gene Name	EntrezGene
1	CDC25A	cell division cycle 25 homolog A (S. pombe)	993
2	CDC20	cell division cycle 20 homolog (S. cerevisiae)	991
3	DBF4	DBF4 homolog (S. cerevisiae)	10926
4	CDC6	cell division cycle 6 homolog (S. cerevisiae)	990
5	CCNB1	cyclin B1	891
6	MCM7	minichromosome maintenance complex component 7	4176
7	CDK1	cyclin-dependent kinase 1	983
8	CCNA2	cyclin A2	890
9	PCNA	proliferating cell nuclear antigen	5111
10	GADD45A	growth arrest and DNA-damage-inducible, alpha	1647
11	MAD2L1	MAD2 mitotic arrest deficient-like 1 (yeast)	4085
12	MCM8	minichromosome maintenance complex component 8	84515
13	ATM	ataxia telangiectasia mutated	472
14	CDKN1B	cyclin-dependent kinase inhibitor 1B (p27, Kip1)	1027
15	BUB1	budding uninhibited by benzimidazoles 1 homolog (yeast)	699

16	CCNB2	cyclin B2	9133
17	CDK6	cyclin-dependent kinase 6	1021
18	CDKN2A	cyclin-dependent kinase inhibitor 2A	1029
19	ORC6	origin recognition complex, subunit 6	23594
