# Supplementary figures and images for: p21 maintains senescent cell viability under persistent DNA damage response by restraining JNK and caspase signaling
Source: EMBO J. 2017 Jun 12;36(15):2280–95. doi: 10.15252/embj.201695553 (PMC5538795; doi:10.15252/embj.201695553)

Appendix Figure S1E - original blots

S1E

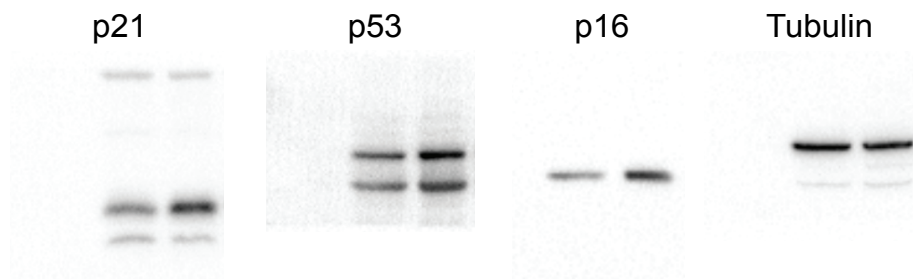

Supplement: Supplementary file 2 — Source Data for Appendix [file EMBJ-36-2280-s006.zip › Source_data_S1E/Original_blots_S1E.pdf]

Appendix Figure S2D - original blots

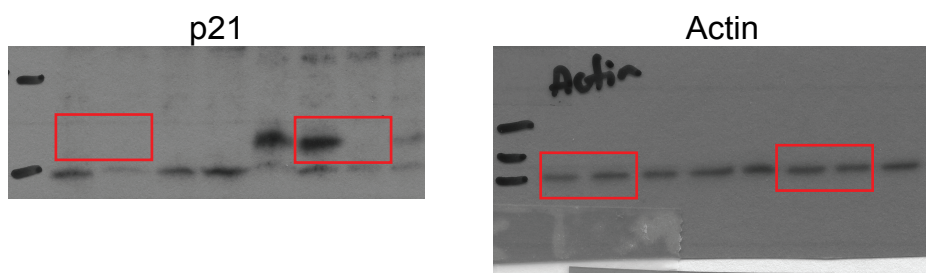

Supplement: Supplementary file 2 — Source Data for Appendix [file EMBJ-36-2280-s006.zip › Source_data_S2D/Original_S2D.pdf]

Appendix Figure S9A - original blots

S9A

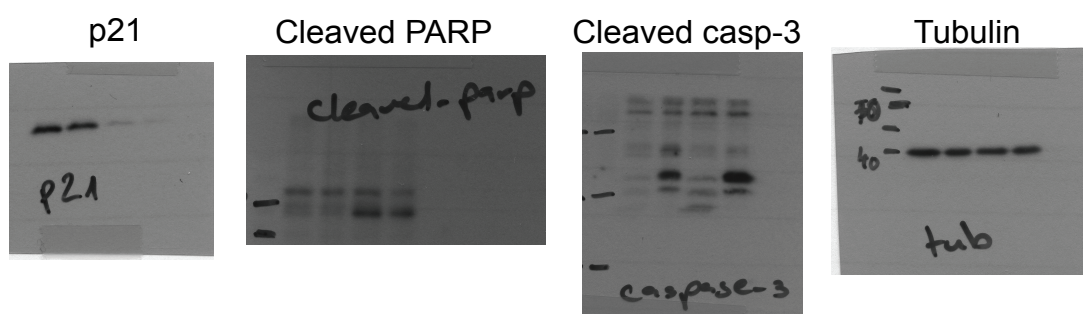

Supplement: Supplementary file 2 — Source Data for Appendix [file EMBJ-36-2280-s006.zip › Source_data_S9A/Original_S9A.pdf]

Figure 3A. - original blots

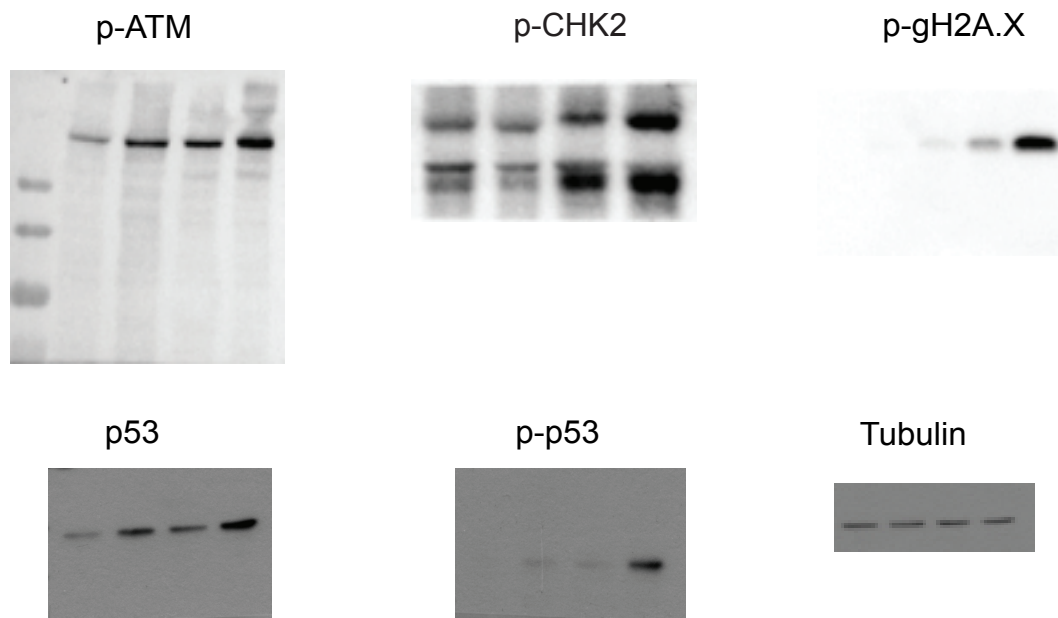

Supplement: Supplementary file 4 — Source Data for Figure 3 [file EMBJ-36-2280-s002.pdf]

Figure 4. - original blots

4A

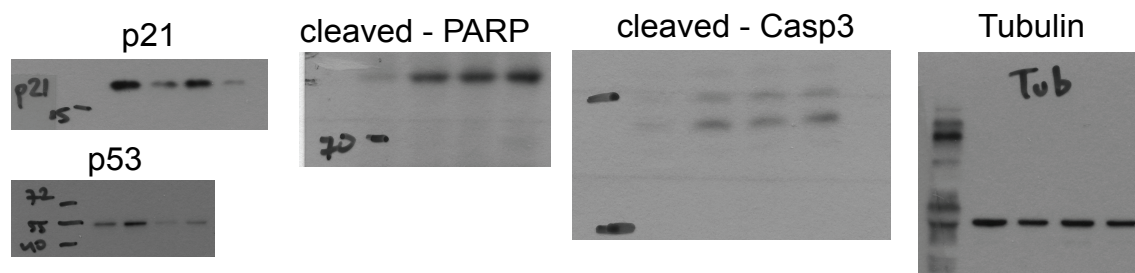

4B

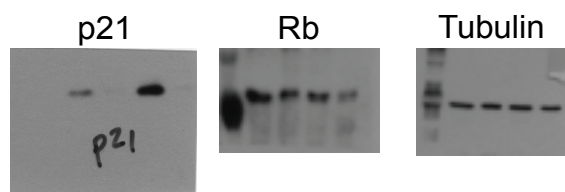

4C

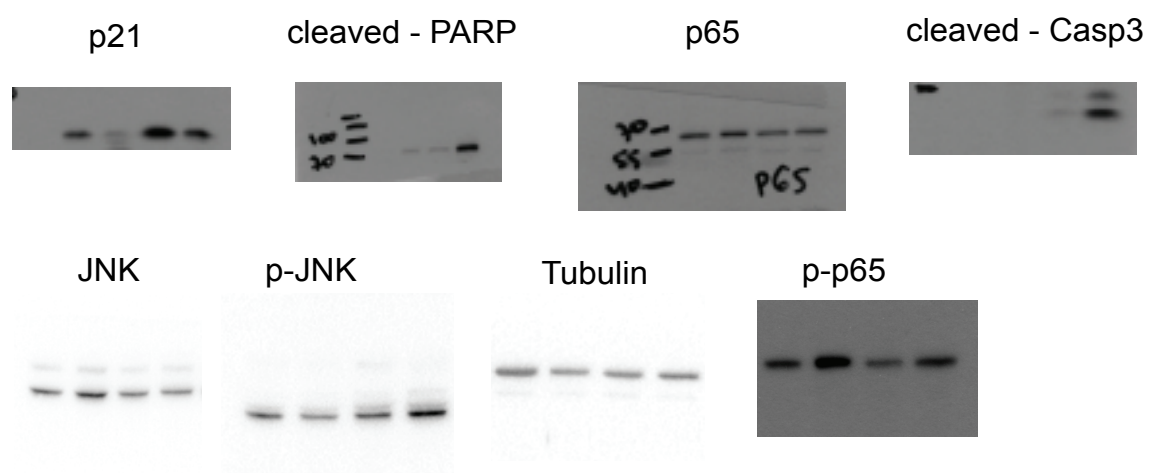

4E

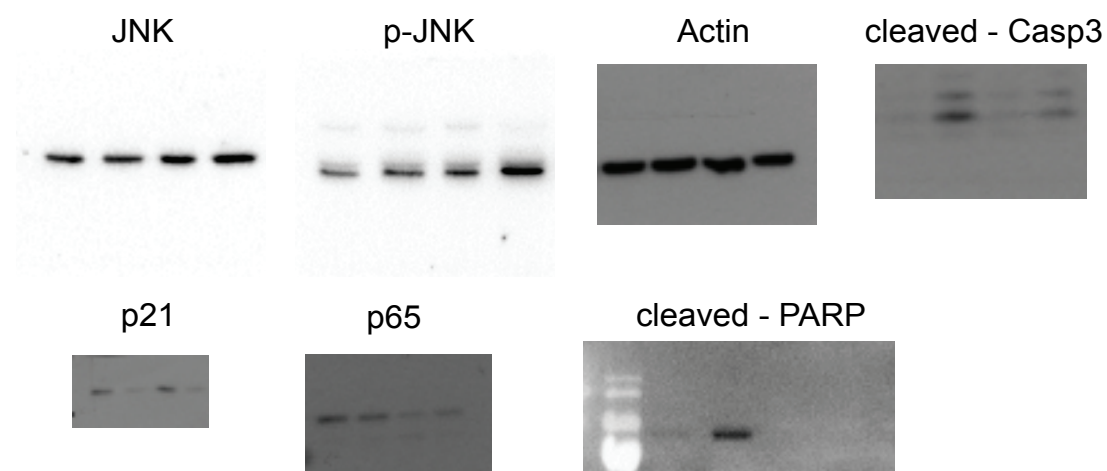

Supplement: Supplementary file 5 — Source Data for Figure 4 [file EMBJ-36-2280-s003.pdf]

Figure 5A. - original blots

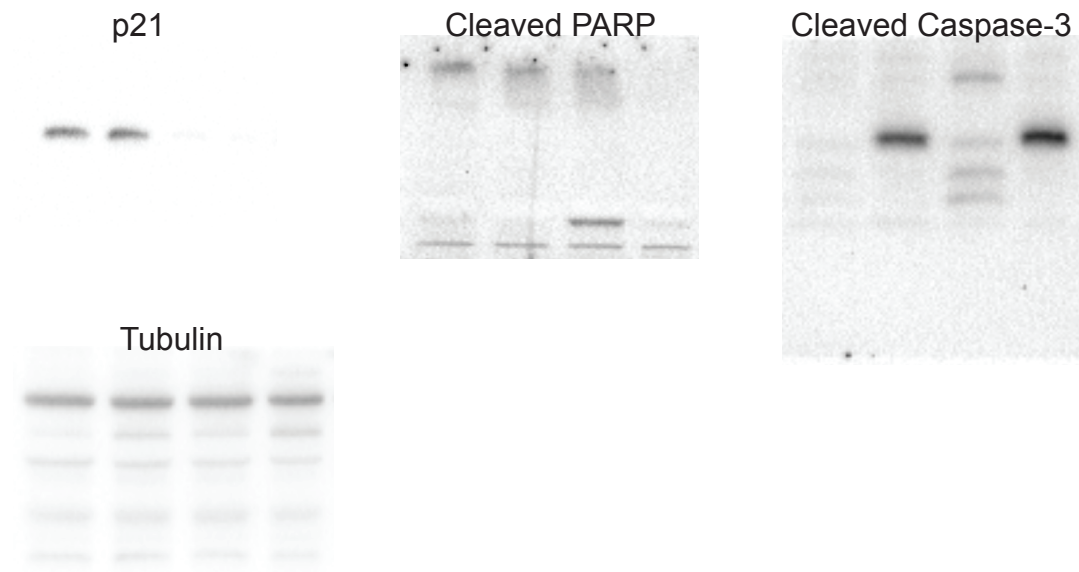

Figure 5. - original blots

5C

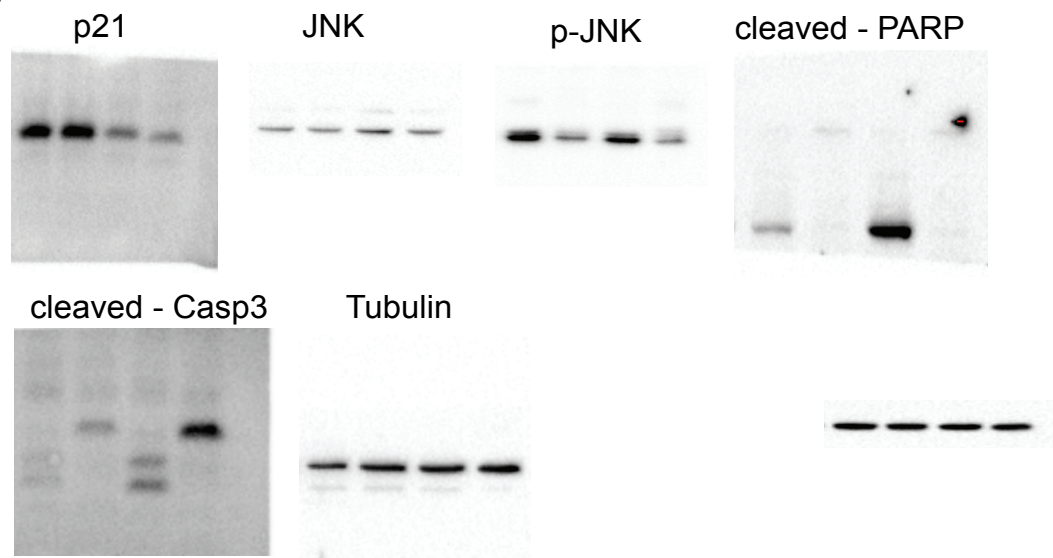

5E

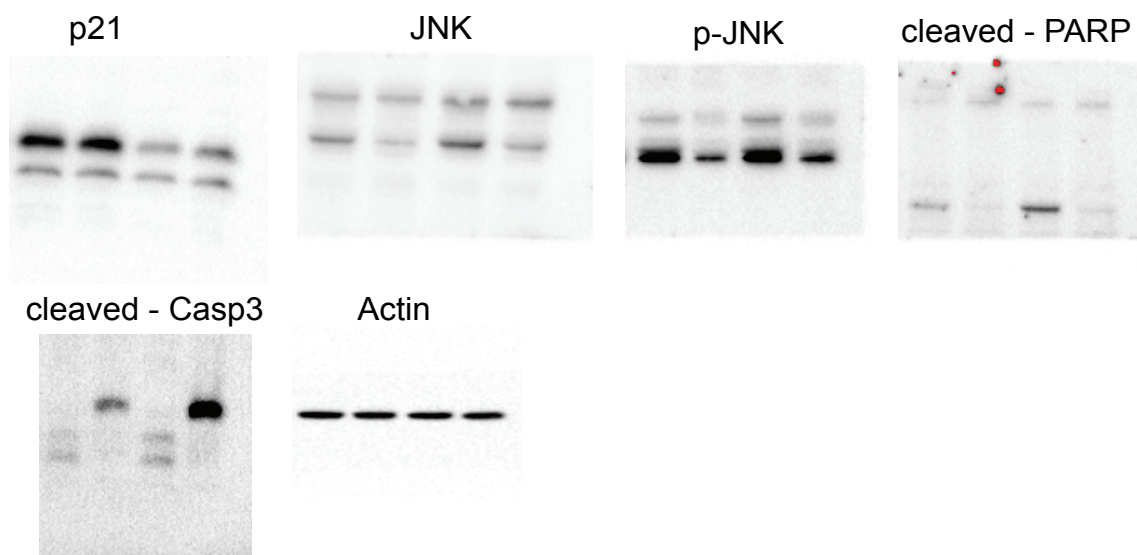

Supplement: Supplementary file 6 — Source Data for Figure 5 [file EMBJ-36-2280-s004.pdf]

Figure 6H - original blots

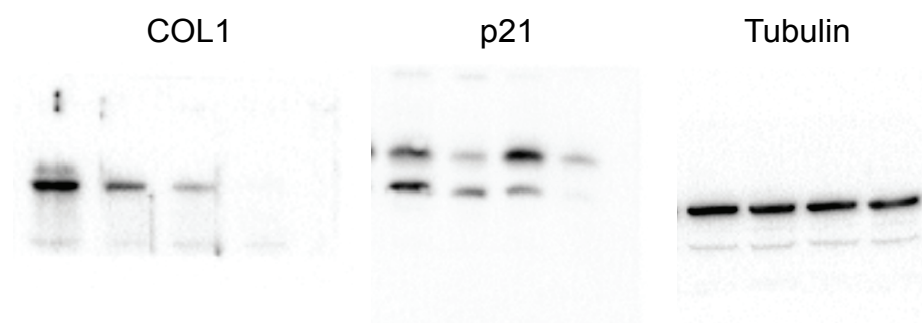

Supplement: Supplementary file 7 — Source Data for Figure 6 [file EMBJ-36-2280-s005.pdf]
